# Immunogenicity and Neutralizing Activity Comparison of SARS-CoV-2 Spike Full-Length and Subunit Domain Proteins in Young Adult and Old-Aged Mice

**DOI:** 10.3390/vaccines9040316

**Published:** 2021-03-29

**Authors:** Ki-Hye Kim, Noopur Bhatnagar, Subbiah Jeeva, Judy Oh, Bo Ryoung Park, Chong Hyun Shin, Bao-Zhong Wang, Sang-Moo Kang

**Affiliations:** Center for Inflammation, Immunity & Infection, Institute for Biomedical Sciences, Georgia State University, Atlanta, GA 30302, USA; kkim39@gsu.edu (K.-H.K.); nbhatnagar1@student.gsu.edu (N.B.); jsubbiah@gsu.edu (S.J.); joh27@gsu.edu (J.O.); bpark9@student.gsu.edu (B.R.P.); cshin@gsu.edu (C.H.S.); bwang23@gsu.edu (B.-Z.W.)

**Keywords:** SARS-CoV-2, spike, neutralizing activity, RBD-hACE2 inhibition, cellular immunity, aged mice

## Abstract

Severe acute respiratory syndrome coronavirus 2 (SARS-CoV-2) continues to be expanding the pandemic disease across the globe. Although SARS-CoV-2 vaccines were rapidly developed and approved for emergency use of vaccination in humans, supply and production difficulties are slowing down the global vaccination program. The efficacy of many different versions of vaccine candidates and adjuvant effects remain unknown, particularly in the elderly. In this study, we compared the immunogenic properties of SARS-CoV-2 full-length spike (S) ectodomain in young adult and aged mice, S1 with receptor binding domain, and S2 with fusion domain. Full-length S was more immunogenic and effective in inducing IgG antibodies after low dose vaccination, compared to the S1 subunit. Old-aged mice induced SARS-CoV-2 spike-specific IgG antibodies with neutralizing activity after high dose S vaccination. With an increased vaccine dose, S1 was highly effective in inducing neutralizing and receptor-binding inhibiting antibodies, although both S1 and S2 subunit domain vaccines were similarly immunogenic. Adjuvant effects were significant for effective induction of IgG1 and IgG2a isotypes, neutralizing and receptor-binding inhibiting antibodies, and antibody-secreting B cell and interferon-γ secreting T cell immune responses. Results of this study provide information in designing SARS-CoV-2 spike vaccine antigens and effective vaccination in the elderly.

## 1. Introduction

Severe acute respiratory syndrome coronavirus 2 (SARS-CoV-2) has been continuing to rapidly spread across the globe [1] since the first outbreak in December 2019 [2,3,4]. SARS-CoV-2 causes pandemic coronavirus disease 2019 (COVID-19), leading to acute respiratory distress syndrome, significant mortality, and lingering symptoms in some individuals after recovery [5]. The global confirmed cases were reported to be over 112 million individuals claiming 2.48 million deaths as of 24 February 2021 [1].

Neutralizing antibodies were shown to provide protection against SARS-CoV-2, indicating a key protective immune correlate [6,7]. The spike (S) glycoprotein trimer is a membrane protein that is consisted of the S1 subunit with the receptor-binding domain (RBD) and the S2 subunit mediating membrane fusion [8,9]. The SARS-CoV-2 S1 RBD domain binds to the host cell receptor, angiotensin-converting enzyme-2 (ACE2), and is the target for 90% of the neutralizing activity in COVID-19 convalescent sera [10]. The S glycoprotein on SARS-CoV-2 is the major target for neutralizing antibodies. Several therapeutic medications, such as neutralizing monoclonal antibodies, hydroxychloroquine, remdesivir, and dexamethasone, have been applied to treat the patients with COVID-19, but the benefits are limited or turned out to be insignificant [11,12,13,14].

Vaccination could be the most effective prophylactic measure to prevent the ongoing COVID-19 pandemic [15,16,17]. Vaccine development was initiated in early January 2020 and has progressed at an unprecedented speed, resulting in licensing few highly effective SARS-CoV-2 vaccines on the market. The mRNA-based SARS-CoV-2 vaccines, BNT162b2 (Pfizer/BioNTech) and mRNA-1273 (Moderna) were recently licensed and approved for vaccination against COVID-19 [18]. Recombinant vectored vaccines, ChAdOxnCoV-19 developed by AstraZeneca/University of Oxford and Sputnik V developed in Russia, were approved for human vaccination. A recombinant full-length S protein nanoparticle vaccine developed by Novavax [19] is now under Phase III clinical trials [15]. Most of these SARS-CoV-2 vaccines, either licensed or advanced to Phase III, are based on the immunity to the full-length S protein. Inactivated SARS-CoV-2 vaccines developed by Sinopharm in China are in clinical Phase III trials [20].

Different platforms of RBD-based SARS-CoV-2 vaccines are in clinical trials and preclinical studies [1,16,17]. Full-length S was shown to be more immunogenic than the S1 subunit in a recent study which used a lentiviral delivery vector expressing S and S1 [21]. A previous study reported superior immunogenicity of SARS-CoV-2 S1 subunit over the RBD domain [22]. In contrast, another study demonstrated that a higher neutralizing titer was induced by RBD than that by S1 and full-length S [23]. A full-length S protein is large (~1300 amino acid residues) and often encounters production difficulties. Vaccine dosage effects by different versions (S, S1, S2) of S protein antigens remain unknown, which is an important parameter in vaccine immunogenicity comparison. In this study, we compared the immunogenicity of full-length S, subunits S1 and S2, and investigated the vaccine dosage and adjuvant effects in young adult and aged mice. Furthermore, inactivated virus SARS-CoV-2 was included for comparison. The results in this study support current strategies of SARS-CoV-2 vaccination and provide informative insights into designing and selecting the S immunogens and effective vaccination in the elderly population.

## 2. Materials and Methods

### 2.1. Recombinant Proteins and Reagents

SARS-CoV-2 different recombinant S and receptor proteins were obtained from Sino Biologicals (Wayne, PA, USA): Full-length S (S1–S2) ectodomain amino acid (aa) residues 16-1213 (40589-V08B1, 134.36 kDa, expressed in baculovirus-insect cells), S1 subunit (aa 16-685) with RBD domain (40591-V08H, 76.5 kDa, expressed in HEK293 cells); S2 subunit (aa 686-1213) with fusion domain (40589-V08B1, 59.36 kDa, expressed in baculovirus-insect cells); human angiotensin-converting enzyme 2 (hACE2) protein (aa 1-740) fused to Fc tag (10108-H02H, expressed in HEK293 cells). Heat-inactivated (65 °C, 30 min; NR-52286) and gamma-irradiated SARS-CoV-2 (NR-52287), and human embryonic kidney cells expressing hACE2 (HEK-293T-hACE2) were provided from BEI/ATCC resources. The SARS-CoV-2 spike pseudoviral particles were purchased from eEnzyme (Gaithersburg, MD). The monophosphoryl lipid A (MPL) and the saponin QS-21 were purchased from Sigma-Aldrich and Desert King, respectively, and used as AS01-like combined vaccine adjuvant (MPL 1 µg + QS-21 10 µg). AS01 (QS-21 + MPL) liposome adjuvant is licensed and included in herpes Zoster vaccination [24,25,26,27].

### 2.2. Mice Immunization

Female 6–8 weeks old young adult BALB/c mice were purchased from the Jackson Laboratory (Bar Harbor, ME, USA). Aged (15 months old) BALB/c mice from National Cancer Institute (NCI/NIH, Bethesda, MD, USA) or retired 8 to 9 months (M) old BALB/c mice were obtained from Taconic Farms (Hudson, NY, USA) and aged to become 15 M old. Animal experiments were performed under the guidelines of the approved institutional animal care and use committee (IACUC) protocol (A21004). Young adult or 15 M old aged BALB/c mice (*n* = 5–6 per group) were intramuscularly (IM) immunized twice (or three times) at 3- or 4-week intervals with full-length S (S1–S2, 0.8 µg equal to 5.9 nanomoles (nM) in young and aged mice, 4 µg equal to 29.8 nM in aged mice), S1 (0.8 µg 10.5 nM or 4 µg 12.3 nM) and S2 (4 µg equal to 67.4 nM), and inactivated SARS-CoV-2 (0.8 µg prime and 10 µg boost). Adjuvants (MPL + QS-21, 1 µg + 10 µg, respectively) were included in S, S1, S2, and inactivated SARS-CoV-2 vaccination as indicated. Blood samples were collected 2 or 3 weeks after immunization to determine IgG binding and neutralizing antibodies.

### 2.3. Pseudovirus Neutralization Assay

The neutralizing antibody titers were determined by a pseudovirus-based assay as previously described [28]. Briefly, immune sera were heat-inactivated at 56 °C for 30 min prior to neutralization assays. Lentiviruses pseudotyped with SARS-CoV-2 S were pre-incubated with an equal volume of serially diluted immune sera for 1 h at room temperature (RT), then virus-antibody mixtures were added to HEK293T-hACE2 cells in a 96-well plate. After a 2 h incubation, the inoculum was replaced with fresh medium. Cells were lysed 48 h later and luciferase activity was measured using luciferin-containing substrate (Promega, Durham, NC, USA). Controls included cell-only control, virus without any antibody control, and positive control sera.

### 2.4. hACE2 Receptor Binding and Inhibition Assay

To confirm the binding ability of recombinant proteins to receptor hACE2 protein expressed from HEK293 cells, the 96-well plates were coated with 0.8 or 2 µg of S (S1–S2) and S1 protein at 4 °C. One day later, serially diluted soluble hACE2 (0.5–2 µg/mL) in phosphate buffered saline with tween-20 (PBST) was added to the plates which were incubated for 2 h at room temperature (RT) after blocking for 1 h and washing the precoated plates. The binding amounts were determined by horseradish peroxidase (HRP)-conjugated anti-human IgG (Southern, Biotech, Birmingham, AL, USA) and 3,3′,5,5′-tetramethylbenzidine (TMB, eBioscience, San Diego, CA, USA).

To detect whether the immune sera can block the binding between SARS-CoV-2 RBD and hACE2, the receptor binding inhibition activity was performed as previously described [29]. Briefly, the recombinant S1 (RBD) protein (400 ng/mL per well) was captured on ELISA plates. Boost immune sera at three-fold dilutions were added onto the plates. After 2 h incubation at RT, the plates were washed and applied with hACE2-Fc (0.5 µg/mL) in PBST at RT for 2 h. The inhibition activity was determined using anti-human IgG-HRP. Pre-immunized sera (naïve) were used as a negative control.

### 2.5. Enzyme-Linked Immunosorbent Assay (ELISA)

Antigen-specific antibody responses were determined from immune sera collected after immunization by ELISA. Briefly, serially diluted immune sera were applied to a 96-well plate precoated with full-length S, S1, and S2 protein (200 ng/mL per well). The levels of antibodies were determined by HRP-conjugated anti-mouse IgG, IgG1, IgG2a (Southern Biotech) and TMB (eBioscience).

### 2.6. Enzyme-Linked Immunospot Assay (ELISpot)

To determine antibody-secreting cells (ASC) specific for full-length S protein, spleen cells were prepared from mice with boost immunization and applied onto 96-well ELISpot plates precoated with full-length S protein (200 ng/mL per well). After 48 h, the antibody responses were determined by anti-mouse IgG and IgG isotypes (IgG1, IgG2a). For cytokine-secreting cells, splenocytes (10^6^ cells per well) were cultured on 96-well ELISpot plates precoated with anti-mouse IFN-γ capture monoclonal antibody (mAb, BD Biosciences, San Diego, CA, USA) in the presence of pooled S peptides (5 µg/mL, BEI resources) and full-length S protein (1 µg/mL). The cytokine-secreting cell spots were developed with biotinylated anti-mouse IFN-γ detection antibody and alkaline phosphatase-labeled streptavidin (BD Pharmingen, San Diego, CA, USA). The spots were visualized with a 3,3′-diaminobenzidine substrate and counted by an ELISpot reader (BioSys, Miami, FL, USA).

### 2.7. Flow Cytometry

For T cell immune responses specific for virus antigens, the splenocytes were harvested from boost immunized mice and were in vitro stimulated with pooled S peptides and full-length S protein for 24 h. The lymphocytes were stained with anti-mouse CD4 (RM4-5, eBioscience), CD8 (53-6.7, eBioscience), and CD3 (17A2, BioLegend) monoclonal antibodies. A BD Cytofix/Cytoperm^TM^ Plus kit was used to fix and permeabilize cells prior to staining with anti-mouse IFN-γ (XMG1.2, eBioscience) monoclonal antibody. All samples were analyzed on a Becton-Dickinson LSR-II/Fortessa flow cytometer (BD Biosciences) and analyzed using Flowjo software (Tree Star Inc., Ashland, OR, USA).

### 2.8. Statistical Analysis

All results are presented as mean ± standard errors of the mean (SEM). The statistical significance for all experiments was performed by one- or two-way analysis of variance (ANOVA). Prism software (GraphPad Software, Inc., San Diego, CA, USA) was used for all data analysis. The comparison used to generate a *p* value is indicated by horizontal lines (*; *p* < 0.05, **; *p* < 0.01, ***; *p* < 0.001).

## 3. Results

### 3.1. SARS-CoV-2 Full-Length Spike S1–S2 Protein Is More Immunogenic Than S1 Subunit Protein

**Figure 1:**
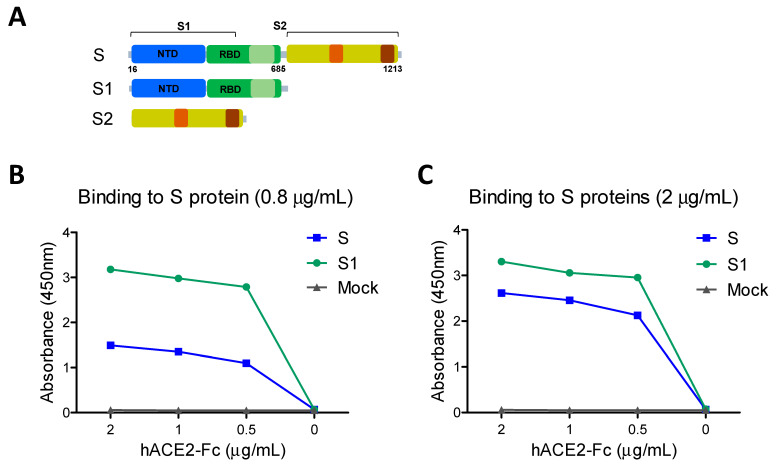
SARS-CoV-2 full-length spike (S) ectodomain and subunit proteins and receptor binding activities. (**A**) Full-length S (S1–S2) ectodomain contains aa residues 16-1213, S1 subunit aa 16-685 (green), and S2 subunit aa 686-1213. NTD: N-terminal domain (blue), RBD: receptor binding domain. (**B**,**C**) The receptor binding properties were determined using serially diluted soluble hACE2-Fc (0.5–2 µg/mL) on the 96-well plates precoated with 0.8 µg (**B**) or 2 µg (**C**) of S (S1–S2) and S1 subunit proteins. Due to different molecular masses of S and S1 proteins despite the same concentration, molarity in nanomoles (nM) is indicated for each protein coated.

It is important to determine the functional integrity of vaccine candidates and their correlation with immunogenicity. The receptor hACE2 binding activity of the full-length ectodomain spike (S: S1–S2) was compared with that of the S1 subunit protein containing the RBD (Figure 1). The full-length S coated ELISA plates at low (0.8 µg/mL, 5.95 nM, Figure 1B) and high (2 µg/mL, 14.89 nM, Figure 1C) concentrations showed less hACE2 binding reactivity values than those in the S1 subunit plates (10.46 and 16.1 nM, respectively). These results suggest that the S1 subunit containing RBD has similar or slightly higher receptor binding activity against hACE2 compared to the full-length S, possibly due to higher molarity.

**Figure 2:**
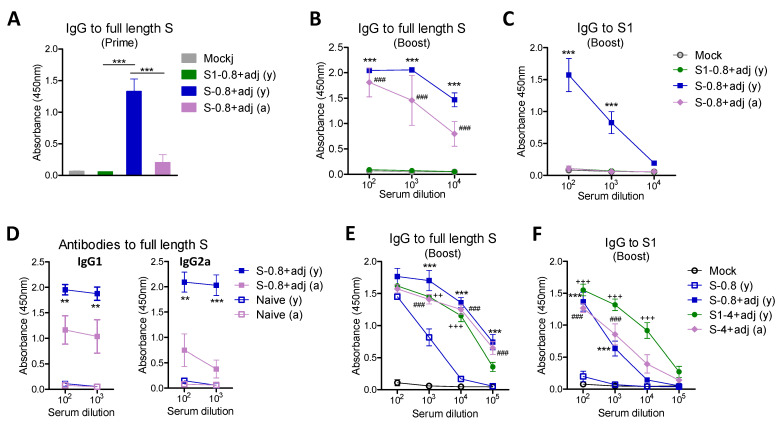
Full-length S protein is more immunogenic than S1 subunit protein. Young and aged BALB/c mice (*n* = 6 to 8) were intramuscularly (IM) immunized twice with S1 (0.8 µg) and S (0.8 µg or 4 µg) in presence of adjuvants (MPL + QS-21, 1 µg + 10 µg), and adjuvant only control (mock). Antigen-specific antibody responses were determined by ELISA. (**A**) IgG specific for full-length S in prime sera (100x dilution) collected at 3 weeks after prime immunization. (**B**,**C**) IgG specific for full-length S and S1 subunit protein in boost sera. Data were compared with mock control. (**D**) Antibodies specific for full-length S. (**E**,**F**) Comparison of low and high dose vaccines inducing IgG antibodies specific for full length S and S1 protein in boost sera. Data were compared between S (0.8 µg) alone without adjuvant and adjuvanted S (0.8 µg, ***; *p* < 0.001) or adjuvanted S1 (4 µg, +++; *p* < 0.001, ++; *p* < 0.01) in young (y) age mice (*n* = 6), and adjuvanted S (4 µg, ###; *p* < 0.001) in old aged (a) mice (*n* = 8). S-0.8 (y): S 0.8 µg vaccination of young adult mice, S-0.8 + adj (y): S 0.8 µg + adjuvant vaccination of young adult mice, S-0.8 + adj (a): S 0.8 µg + adjuvant vaccination of old aged mice, S1-4 + adj (y): S1 4 µg + adjuvant vaccination of young adult mice, S-4 + adj (a): S 4 µg + adjuvant vaccination of old aged mice. Adj: adjuvants (MPL + QS-21, 1 µg + 10 µg). Statistical significance was calculated using one- or two-way ANOVA and a Bonferroni’s multiple-comparison test. Error bars indicate the mean ± standard errors of the mean (SEM). **; *p* < 0.01, ***; *p* < 0.001 compared to mock control.

Initially, we compared the immunogenicity of the full-length ectodomain S (aa#16-1213) protein in comparison with the S1 subunit (aa#16-685) protein (Figure 2). Young adult BALB/c mice (6–8 weeks old, *n* = 5) were intramuscularly (IM) immunized with a low dose (0.8 µg) of S (S1–S2) or S1 protein in the presence of AS01-like adjuvant (QS-21 + MPL) at weeks 0 and 4. To determine the aging effects on immunogenicity, 15 months (M) old-aged mice (*n* = 5) were immunized with the same low dose (0.8 µg) of S plus QS-21 + MPL adjuvant. At 3 weeks after prime, high levels of IgG antibodies specific for S protein were induced in the S young adult group whereas no or low levels of S specific IgG antibodies were observed in the young adult S1 and aged S (S1–S2) groups (Figure 2A). At 3 weeks after boost, the aged S group induced substantial levels of S-specific IgG antibodies although at lower levels compared to those in the young adult S group (Figure 2B). Substantial levels of S1-specific IgG antibodies were induced in the young adult age S group but not in the aged mouse S group with a 0.8 µg dose (Figure 2C). The old-aged mice vaccinated with a 0.8 µg dose showed more defects in inducing IgG2a than IgG1 isotype antibodies specific for full-length S compared to those of the young adult S group (Figure 2D). These results suggest that SARS-CoV-2 full-length spike protein is more immunogenic than the S1 subunit protein and higher doses of protein vaccines might be needed to induce comparable IgG antibodies in aged populations.

### 3.2. Aged Mice and S1 and S2 Subunit Domain Proteins Need a High Vaccine Dose for Effective Induction of SARS-CoV-2 Spike-Specific IgG Antibodies

Since a low dose (0.8 µg) of S1 subunit protein vaccine was not immunogenic even in young adult mice and the full-length 0.8 µg S in aged mice, we determined whether a high dose would be required for effective induction of SARS-CoV-2 spike-specific IgG antibodies for aged mice and subdomain S1 protein (Figure 2E,F). The aged mouse group that was immunized with a high dose (4 µg) of S protein plus adjuvant induced comparable levels of S and S1 specific IgG antibodies as those in the low dose (0.8 µg) S young adult group (Figure 2E,F). Moreover, to determine the adjuvant effects, the same 0.8 µg dose S full-length with and without QS-21 + MPL adjuvant was included in the young adult age mouse groups (Figure 2E,F). The S (S1–S2) group without adjuvant induced lower levels of S-specific IgG and lowest levels of S1 (RBD) specific IgG antibodies, compared to those in S vaccination with adjuvant. The adjuvanted S1 (4 µg) young adult mice showed the highest level of S1 specific IgG antibody responses (Figure 2F). These results indicate that effective induction of S-specific IgG antibody responses was observed by a high vaccine dose (4 µg) of adjuvanted full-length S in aged mice and subunit S1 vaccine in young adult mice. Furthermore, these results support the significant roles of adjuvants in inducing S1 and full-length S specific IgG antibody responses.

### 3.3. Adjuvanted Spike Protein Vaccinations Effectively Induce Subunit S Domain Specific IgG1 and IgG2a Isotype Antibodies

**Figure 3:**
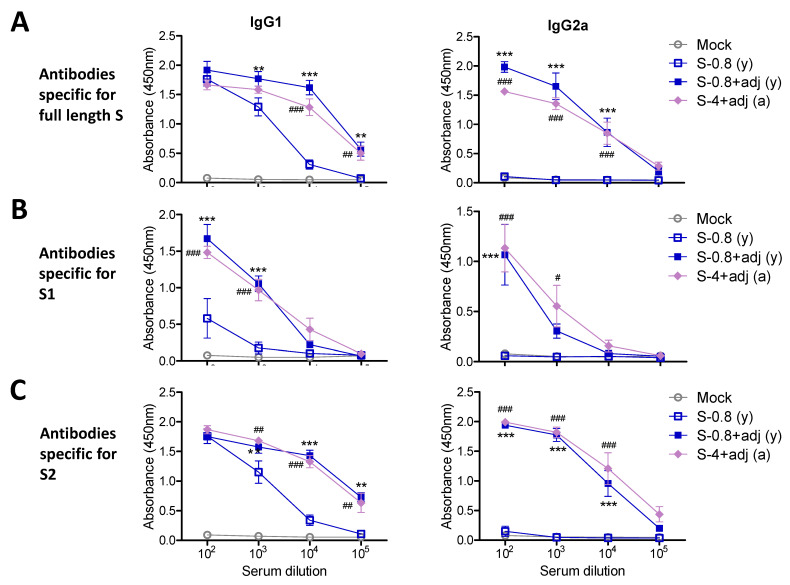
IgG isotype antibody responses to 0.8 µg S vaccination in young age mice and 4 µg S vaccination in old-aged mice. Blood samples were collected from vaccinated young mice (*n* = 6) with either S (S-0.8: 0.8 µg) only or S (0.8 µg) + adjuvant (S-0.8 + adj), adjuvant only mock control (MPL + QS-21, 1 µg + 10 µg), and from aged mice (*n* = 8) with S (4 µg) + adjuvant (S-4 + adj) after boost. (**A**) Full-length S-specific IgG isotype antibodies in boost sera. (**B**) S1 subunit protein-specific IgG1 and IgG2a isotype antibodies after boost. (**C**) S2 subunit protein-specific IgG isotype antibodies in boost sera. (y): indicates young adult age mice in the group (*n* = 6), (a): indicates old aged mice in the group (*n* = 8). Statistical significance was calculated using two-way ANOVA and a Bonferroni’s multiple-comparison test. Error bars indicate the mean ± SEM. **; *p* < 0.01, ***; *p* < 0.001, #; *p* < 0.05, ###; *p* < 0.001, ##; *p* < 0.01. Results were compared to no adjuvanted S group.

**Figure 4:**
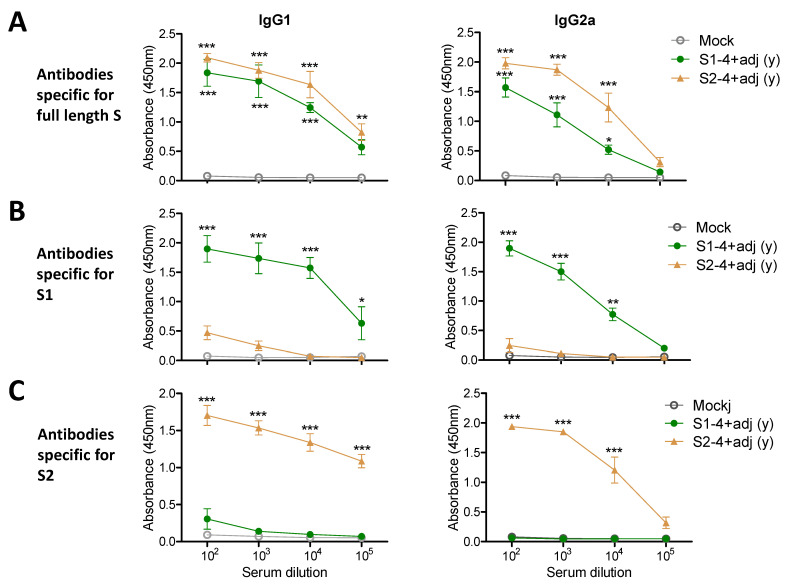
IgG isotype antibody responses to S1 or S2 (4 µg) vaccination in young adult age mice. Young BALB/c mice (*n* = 6) were IM immunized twice with 4 µg of S1 (S1-4 + adj) and S2 (S2-4 + adj) with adjuvants (MPL + QS-21, 1 µg + 10 µg) and adjuvant only (mock). Antigen-specific IgG isotype antibody responses were determined in boost sera by ELISA. IgG1 and IgG2a isotype antibodies specific for full length S protein (**A**), for S1 subunit protein (**B**), for S2 subunit protein (**C**). (y): indicates young adult age mice in the group. Statistical significance was calculated using two-way ANOVA and a Bonferroni’s multiple-comparison test. Error bars indicate the mean ± SEM. *; *p* < 0.05, **; *p* < 0.01, ***; *p* < 0.001 and compared with the mock control.

In the additional experimental setting to determine IgG isotypes, the groups of young adult mice (*n* = 5) were IM prime boost immunized with full-length S (0.8 µg) +/- adjuvant QS-21 + MPL (Figure 3), S1 subunit (4 µg) + adjuvant, or S2 subunit (4 µg) + adjuvant (Figure 4). The aged mice (*n* = 5) were IM prime boost immunized with S (4 µg) + adjuvant for comparison (Figure 3). Both adjuvanted 4 µg S immunized 15M old-aged mice and 0.8 µg S immunized young adult mice showed similar levels of IgG1 and IgG2a antibodies specific for full length S, subunits S1 and S2 (Figure 3). The unadjuvanted S immunized young adult mice induced lower levels of IgG1 antibody for S and S2 (Figure 3A,C), further lower levels of S1 recognizing IgG1 antibody (Figure 3B), and undetectable levels of IgG2a isotype antibody, compared to those in adjuvanted S immunization (Figure 3). It is interesting to note that 4 µg of S2 vaccination induced higher levels of S specific IgG1 and IgG2a antibodies than S1 vaccination (Figure 4A), suggesting that S2 might be more immunogenic than S1. As expected, the adjuvanted 4 µg S1 group induced S and S1 specific IgG1 and IgG2a isotype antibodies whereas the adjuvanted 4 µg S2 group induced S and S2 specific IgG1 and IgG2a isotype antibodies (Figure 4). Overall, a dose of 4 µg S protein was highly immunogenic in aged mice, and 4 µg S1 or S2 proteins were comparably immunogenic in young adult mice. AS01-like adjuvant QS-21 + MPL was effective in enhancing S vaccine specific IgG1 and IgG2a isotype antibodies in young and aged mice.

### 3.4. Vaccination with Adjuvanted S or S1 Proteins Induced Pseudovirus Neutralizing and Receptor Inhibiting Antibodies

**Figure 5:**
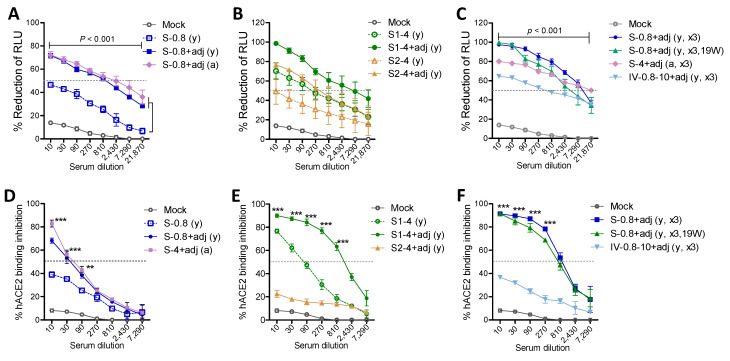
Adjuvanted S and S1 immune sera exhibit high titers of SARS-CoV-2 pseudovirus neutralization and receptor binding inhibition activities. (**A**–**C**) Reduction percentage (%) in relative luminometer units (RLU) as a measure of luciferase activity for SARS-CoV-2 spike pseudotyped lentivirus infection in HEK293 cells expressing human ACE2 receptor. Data were obtained from pooled sera (*n* = 6 to 8) with triplicate wells. S-0.8 (y): S 0.8 µg boost sera of young adult mice, S-0.8 + adj (y): S 0.8 µg + adjuvant boost sera of young adult mice, S-0.8 + adj (a): S 0.8 µg + adjuvant boost sera of old aged mice, S1-4 (y): S1 4 µg boost immune sera of young adult mice, S1-4 + adj (y): S1 4 µg + adjuvant boost immune sera of young adult mice, S2-4 (y): S2 4 µg boost immune sera of young adult mice, S2-4 + adj (y): S2 4 µg + adjuvant boost immune sera of young adult mice, S-0.8 + adj (y, x3): S 0.8 µg + adjuvant 2nd boost immune sera of young adult mice, S-0.8 + adj (y, x3, 19W): S 0.8 µg + adjuvant immune sera collected at week 19 post 2nd boost of young adult mice, S-4 + adj (a, x3): S 4 µg + adjuvant 2nd boost sera of old aged mice. IV-0.8-10 + adj (y, x3): inactivated adjuvanted SARS-CoV-2 vaccination in young age mice (prime 0.8 µg of heat-inactivated and gamma-irradiated virus, 2 times boost with 10 µg inactivated adjuvanted SARS-CoV-2 of heat-inactivated and gamma-irradiated virus). Adj: adjuvants (MPL + QS-21, 1 µg + 10 µg). Mock: sera from mice with adjuvant (MPL + QS-21, 1 + 10 µg) only. (**D**–**F**) ACE2 receptor binding inhibition titers in pooled immune sera (*n* = 6–8) with triplicate wells. Inhibition percentage (%) of hACE2 binding to RBD was measured after incubation with serially diluted immune sera in the plate precoated with hACE2 protein. Immune sera of groups are the same as in (**A**–**C**). Statistical significance was calculated using two-way ANOVA and a Bonferroni’s multiple-comparison test. Error bars indicate the mean ± SEM. **; *p* < 0.01, ***; *p* < 0.001 compared to the mock or no adjuvant control.

Induction of neutralizing antibodies after vaccination is considered a major correlate with protection against SARS-CoV-2. Boost sera from the adjuvanted 0.8 µg S-immunized young mice and 4 µg-S immunized old mice showed similarly high levels of SARS-CoV-2 pseudovirus neutralizing antibody titers (810) of 50% reduction (Figure 5A). Boost sera from the unadjuvanted 0.8 µg S young adult mice could not neutralize SARS-CoV-2 pseudovirus at a meaningful level (Figure 5A). Adjuvanted 4 µg S1 vaccination induced high neutralizing titers of approximately 2430–7290 in boost sera, whereas the adjuvanted 4 µg S2 group induced low neutralizing titers of 270, similar to the unadjuvanted S1 group (Figure 5B). The second boost with 0.8 µg S was found to increase neutralizing titers to a range of 7290, which was retained for over 4 months (Figure 5C). Furthermore, the 15M aged mice with secondary boost S induced high neutralizing titers (~7290) (Figure 5C). Inactivated SARS-CoV-2 (0.8 µg prime, 10 µg boost 2 times) immune sera showed a lower range of neutralizing titers (90 to 270, Figure 5C).

A major mechanism of neutralizing immunity might be antibody mediated interference with binding of the SARS-CoV-2 spike RBD to the hACE2 receptor. Low levels of hACE2 binding inhibition (50%) titers of ~30 were observed with boost sera from 0.8 µg S young and 4 µg aged mice, respectively (Figure 5D). A high titer (over 810) of hACE2 binding inhibition antibodies was induced in adjuvanted 4 µg S1 but not S2 young mice (Figure 5E). Notably, 4 µg S1 vaccination of young adult mice could induce a low level of receptor inhibiting activity (~90) titers even in the absence of adjuvant (Figure 5E). Moreover, a second boost with adjuvanted S (0.8 µg) but not with inactivated SARS-CoV-2 in young adult mice resulted in a high titer (~810) of hACE2 binding inhibition (Figure 5F). These results suggest that vaccination with adjuvanted S or S1 proteins effectively induces high titers of pseudovirus neutralizing and receptor inhibiting antibodies. It is likely that antibodies to the S2 subunit can partially contribute to neutralizing SARS-CoV-2 pseudovirus in a different mechanism.

### 3.5. Adjuvanted Vaccination Enhances S-Specific Cellular Immune Responses

**Figure 6:**
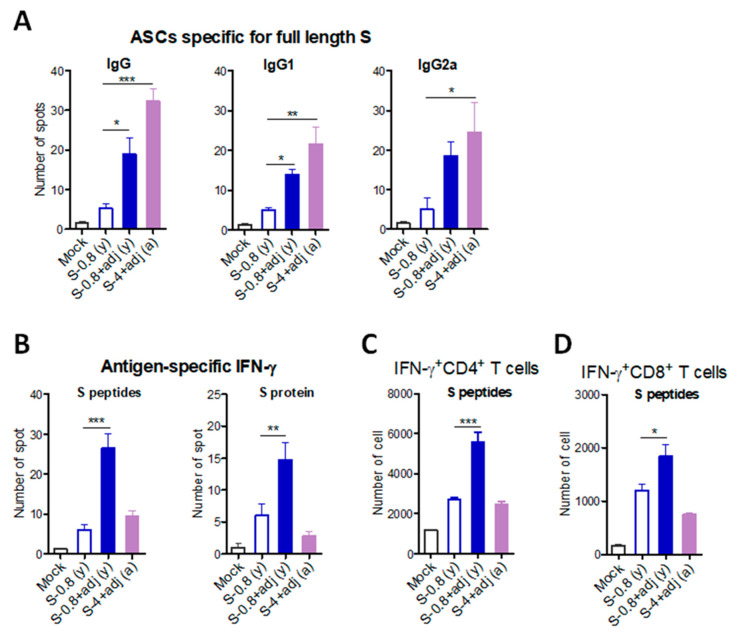
B cell and T cell immune responses to SARS-CoV-2 S vaccination in young adult and old aged mice. To determine cellular immunity, spleen cells were prepared from immunized young adult (*n* = 6) and old aged mice (*n* = 8). (**A**) Antibody-secreting cells (ASCs) specific for full-length S protein were determined on the ELISpot plate precoated with full-length S protein. (**B**) IFN-γ-secreting cells were analyzed by in vitro stimulation with pooled S peptides or full-length S protein using ELISpot assay. (**C**,**D**). IFN-γ + CD4 and IFN-γ + CD8 T cells were determined by flow cytometry after in vitro stimulation with pooled S peptides and intracellular cytokine antibi staining. S-0.8 (y): S 0.8 µg vaccination of young adult mice, S-0.8 + adj (y): S 0.8 µg + adjuvant vaccination of young adult mice, S-0.8 + adj (a): S 0.8 µg + adjuvant vaccination of old aged mice. Mock: sera from mice with adjuvant (MPL + QS-21, 1 + 10 µg) only. Statistical significance was calculated using one-way ANOVA and a Dunnett’s multiple-comparison test. Error bars indicate the mean ± SEM. *; *p* < 0.05, **; *p* < 0.01, ***; *p* < 0.001.

Adjuvanted S vaccination could maintain neutralizing and receptor binding inhibition antibodies for over 4 months (Figure 5C,F). At 19 weeks after boost, S-specific antibody-secreting cells (ASCs) in splenocytes were determined (Figure 6A). S-specific IgG, IgG1, and IgG2a ASC responses were induced at significantly higher levels in spleen cells from adjuvanted S vaccinated young (y) adult and aged (a) mice, compared to those from unadjuvanted S only vaccinated mice (Figure 6A). IFN-γ producing splenocytes were also determined after boost. Upon in vitro stimulation of splenocytes with S peptides or S protein, IFN-γ secreting cell spots were detected at the highest level in the adjuvanted S vaccinated young adult mice (Figure 6B). The aged mice with adjuvanted S vaccination induced IFN-γ secreting cell spots at low levels as observed in the young adult mice with unadjuvanted S vaccination (Figure 6B). Consistently, IFN-γ + CD4 T and IFN-γ + CD8 T splenocytes were induced at the highest level in the S vaccinated young adult mice (Figure 6C,D). The number of IFN-γ + CD4 T cells were higher than IFN-γ + CD8 T cells. Both adjuvanted S immunized aged mice and unadjuvanted S immunized young adult mice induced similarly moderate levels of IFN-γ + CD4 T and IFN-γ + CD8 T splenocytes, which were higher than those from mock control mice (Figure 6C,D).

## 4. Discussion

SARS-CoV-2 full-length S mRNA and recombinant adenovirus vector vaccines have been recently approved for emergency use authorization for human vaccination. Many other different SARS-CoV-2 vaccine candidates by diverse vaccine modalities are under preclinical and clinical development. It is of high significance to better understand the immunogenic differences of the different subdomains of SARS-CoV-2 spike protein. There are controversial studies reporting the immunogenicity of different spike subunit domains. The first approved use in human vaccination, SARS-CoV-2 mRNA vaccine (BNT162b2), encodes the full-length spike [30]. SARS-CoV-2 RBD encoding mRNA vaccine (BNT162b1) was also assessed in Phase I and II clinical trial studies [15,30]. The immunogenicity of BNT162b1 and BNT162b2 was comparable in healthy individuals [30]. In the overall safety assessments, BNT162b1 RBD mRNA vaccination was associated with a higher incidence and severity of systemic reactions than BNT162b2 full-length S mRNA now on the market for human vaccination [15,30]. A prior preclinical study reported that the 1 μg dose of mRNA-1273 pre-fusion stabilized SARS-CoV-2 S vaccine in lipid nanoparticles induced comparable levels of S-specific antibodies as raised with 1 μg of S trimer in TLR4 (MPL) agonist adjuvant in mice [31]. We compared the immunogenicity of full-length S and subunit S1 at a low dose (0.8 μg) in AS01-like adjuvant and found that S was more immunogenic than S1 in inducing spike-specific IgG antibodies. With 4 μg dose vaccination, S1 subunit was highly effective in inducing S-specific binding IgG, neutralizing and receptor-binding inhibiting antibodies. A second boost strategy with a low dose (0.8 μg) S was also highly effective in further enhancing neutralizing antibodies, which lasted for at least 4 months. Consistent with this study, recent studies reported that modified vaccinia Ankara and live-attenuated YF17D vectors expressing SARS-CoV-2 S but not S1, induced strong neutralizing antibody responses in mice [32] and hamster animals [33], respectively. A lentiviral vector vaccine expressing full-length S was more immunogenic in mice than the lentivirus expressing the S1 subunit [21]. The SARS-CoV-2 S DNA vaccine was also moderately more effective in inducing neutralizing antibodies than S1 or RBD DNA vaccine in monkeys [7]. S1 subunit protein was shown to be superior to RBD only in inducing neutralizing antibodies as a SARS-CoV-2 subunit vaccine in mice immunized with 10 μg dose vaccine in alum adjuvant, but full-length S was not included for comparison in this previous study [22]. In contrast, another study reported comparable binding IgG antibodies and a higher neutralizing titer by 50 μg SARS-CoV-2 RBD protein mixed with Emulsigen adjuvant, compared to 50 μg SARS-CoV-2 S1 in rabbits, both of which were more effective in inducing S specific IgG and neutralizing antibodies than full-length S [23]. Vaccine doses, animal species, and adjuvants are the factors that can contribute to the differential outcomes among the different studies although it is unclear about these differing results in comparing immunogenic properties of the S and its subunit domains.

A recent study reported that the glycosylation patterns were found to be similar on the SARS-CoV-2 spike (S) ectodomain proteins expressed in insect and human cells [34]. Du et al. (2009) compared the immunogenicity and protective immunity of recombinant SARS-CoV RBD proteins expressed in mammalian cells, insect cells, and *Escherichia coli* [35]. Intact conformation and authentic antigenicity were retained in all recombinant RBD proteins expressed in mammalian cells, insect cells, and *E. coli* [35]. Both recombinant SARS-CoV RBD proteins expressed in 293T mammalian and Sf9 insect cells maintained similar immunogenicity to induce RBD-specific antibodies in vaccinated mice, which was higher than that produced in *E. coli* [35]. Mammalian 293T cell-expressed RBD protein vaccination induced higher levels of neutralizing antibodies than those induced by Sf9 insect or *E. coli* expressed RBD [35]. Therefore, these prior studies suggest that the low immunogenicity of the S1 protein is not because of the mammalian expression system compared to the insect cell expressed full-length S protein but rather a difference in immunogenic conformation between the subunit S1 and full-length S.

The presentation of RBD in nanoparticles was reported to be effective in inducing neutralizing antibodies, although the RBD itself is poorly immunogenic as a monomer [36,37]. Nonetheless, RBD-specific immunity alone would not provide protection against distantly related viruses due to substantial sequence variation among the different coronavirus S RBDs and its limited T cell epitopes [7]. The S2 subunit domain is known to have the sequence and structural conservation among the different coronaviruses and conserved epitopes for potential neutralizing antibodies [8,9]. We found that S2 was as immunogenic as S1 and could induce a low level of neutralizing antibodies independent of hACE2 binding inhibition, which might provide advantages in broadening the protection against viruses with mutations in RBD. Whereas S and S1 vaccination induced higher levels of neutralizing antibodies correlating with titers of hACE2 binding inhibition. Meanwhile, the immunogenicity of inactivated SARS-CoV-2 to induce neutralizing antibodies was lower than the S or S1 subunit, although further studies are needed for this conclusion.

AS01 (QS-21 +MPL) liposome adjuvant is approved for use in herpes Zoster vaccination (shingles, recommended for ≥50 years old) [24,25,26,27]. The effects of AS01-like adjuvant were found to be significant on enhancing Th1 type-IgG2a isotype and S1-specific IgG antibodies, neutralizing and hACE2 binding inhibition activity titers to S vaccination in young and aged mice. In addition, cellular responses of S-specific IgG secreting cells, and IFN-γ+ CD4 and CD8 T cells were significantly enhanced by AS01-like adjuvant in SARS-CoV-2 S vaccination. In contrast, alum adjuvanted SARS-CoV-2 S subunit vaccines were demonstrated to induce Th2-biased immune responses [22]. Overall, adjuvanted SARS-CoV-2 S vaccination is expected to enhance protective IgG and cellular immune responses to S1 and S2 domains in young and elderly populations.

## 5. Conclusions

This study investigated the immunogenic properties of SARS-CoV-2 full-length spike ectodomain (S: S1–S2) in young adult and old-aged mice, S1 with RBD, S2 with fusion domain, and the effects of AS01-like adjuvant (QS-21 + MPL). At a low dose (0.8 µg), full-length S was more immunogenic than the S1 subunit domain. With a 4-µg vaccine dose, S1 was highly effective in inducing neutralizing and receptor-binding inhibition antibodies, although both S1 and S2 subunit domain vaccines were similarly immunogenic. Aged mice required a high vaccine dose (4 µg) of S for effective induction of SARS-CoV-2 spike-specific IgG antibodies. Adjuvant was required to effectively induce IgG1 and IgG2a isotypes, neutralizing antibodies, and ASC and IFN-γ T cell immune responses. Results of this study provide insights into designing SARS-CoV-2 spike vaccine antigens.
